# From a stone to rupture: calyceal rupture secondary to obstructive uropathy

**DOI:** 10.1002/ccr3.1540

**Published:** 2018-04-25

**Authors:** Babikir Kheiri, Ismail Kazmi, Seetharamprasad Madala, Emad Abu Sitta

**Affiliations:** ^1^ Internal Medicine Department Hurley Medical Center/Michigan State University Two Hurley Plaza, Ste 212 Flint 48503 Michigan

**Keywords:** Calyceal rupture, obstructive uropathy, ureteric stone

## Abstract

Renal collecting system rupture is a rare manifestation of obstructive uropathy. The majority of cases are attributed to ureteric calculi and extrinsic ureteric compression. The optimal diagnostic image is contrast‐enhanced CT scan with delayed phase protocol. Management is usually conservative and generally depends on the etiology, urinomas size, and the presence or absence of kidney failure or infections.

## Quiz Question: What is the Diagnosis and the Management?

**Figure 1:**
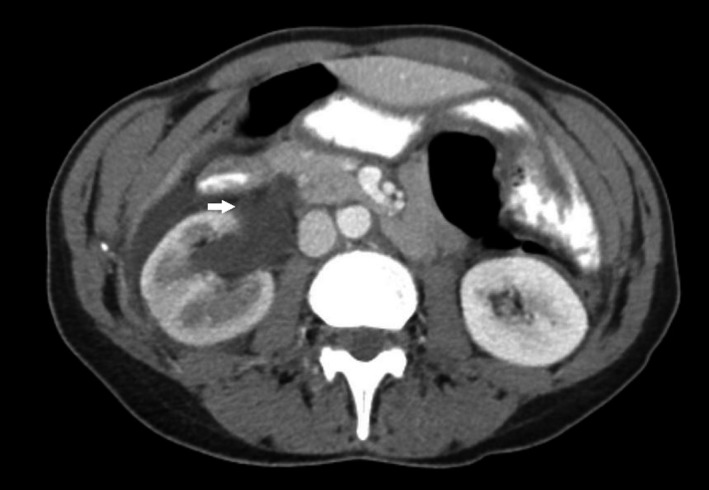
Hydroureteronephrosis and ruptured calyx (arrow).

A 52‐year‐old Caucasian woman presented to the emergency department with acute‐onset right lower abdominal pain for 1 day. She reported a few days history of fever, chills, urinary frequency, and dysuria. Vital signs were noticeable for a temperature of 38.7°C, regular heart rate of 120 beats/min, blood pressure of 104/62, and respiratory rate of 19. Abdominal examination elicited tenderness to the right costovertebral angle. Physical examination otherwise was unremarkable. Laboratory investigations showed a white cell count (WCC) of 19.6 (Ref: 4.0–10.8 K/UL), neutrophil count of 96% (Ref: 36–75%), hemoglobin of 14.9 (Ref: 12.0–16.0 G/DL), normal kidney and liver function tests, as well as a negative pregnancy test. Urinalysis was positive for nitrite, leukocyte esterase (2+), and blood (3+). Urine microscopy showed WBC of 5–10/high‐power field and RBC of 40–60/HPF. Pelvic ultrasound showed normal appearance of uterus, endometrium, and both ovaries. However, a computed tomography (CT) scan of the abdomen showed a 7 mm calculus at the ureterovesical junction causing right hydroureteronephrosis and ruptured calyx (Fig. 1). She was started on intravenous fluids and ceftriaxone. Blood and urine culture grew *Klebsiella pneumoniae*. She underwent right double J‐stent insertion and had successful lithotripsy 2 weeks later.

Calyceal rupture is a rare manifestation of obstructive uropathy 1. In our case, the cause of calyceal rupture is credited to distal ureteric stone. The mechanism of calyceal rupture is assumed to be related to the increased pressure 2. Although the management of small urinoma is generally conservative, obstructive calculi may require ureteral stent and lithotripsy 1. In contrast, larger urinoma may require percutaneous drainage and nephrostomy catheters 1.

## Conflict of Interest

None declared.

## Authorship

BK: designed, planned, and wrote the manuscript and did the literature review. IK: designed, planned, and revised manuscript. SM: designed, planned, and revised the manuscript, and prepared the photographs. EA: designed, planned, and revised the manuscript and selected the photographs.
